# Prevalence, value and food marketing methods of food-related sponsorship and contracts in Canadian recreation and sport facilities

**DOI:** 10.1017/S1368980025101699

**Published:** 2026-03-27

**Authors:** Rachel Joyce Lian Prowse, Melanie Warken, Trudy Tran, Dana Lee Olstad, Sara FL Kirk, Kim D. Raine, Erin Hobin

**Affiliations:** 1 Faculty of Medicine, Memorial University of Newfoundland, Canada; 2 Department of Community Health Sciences, Cumming School of Medicine, University of Calgary, Canada; 3 Faculty of Health, Dalhousie University, Canada; 4 School of Public Health, University of Alberta, Canada; 5 Health Promotion, Chronic Disease, and Injury Prevention, Public Health Ontario, Canada

**Keywords:** Food marketing, Food, Nutrition, Children, Sport, Sponsorship, Marketing communications

## Abstract

**Objective:**

To examine the prevalence, financial value and marketing leveraging methods of food sponsorship agreements and food service contracts in Canadian recreation and sport facilities (RSF).

**Design:**

Cross-sectional survey using descriptive analysis. RSF managers and directors reported the number, value and types of marketing leveraging methods used in food-related sponsorship agreements and food service contracts.

**Setting:**

Publicly funded RSF in nine Canadian provinces that provide indoor sport programming for children and youth.

**Participants:**

Eighty-six RSF representatives completed the survey (response rate: 73·9 %). Most facilities were municipally owned and located in urban settings; over 70 % served children under 13 years of age.

**Results:**

Food sponsorship agreements and food service contracts were reported by 36·5 % and 65·5 % of RSF, respectively. Financial donations were included in 88·6 % of sponsorship agreements and 27·4 % of contracts. Sponsors contributed a median of 25·0 % (IQR: 13·9–83·3 %) of total sponsorship income, with a median annual donation per sponsor of $500 (IQR: $288–$1375). Nearly all agreements and contracts included at least one food marketing leveraging method. Branded signage was the most common in sponsorship agreements (64·6 %), while equipment donation was the most common in food service contracts (52·2 %).

**Conclusions and Implications:**

Food sponsorship and service agreements are prevalent in Canadian RSF and include financial and in-kind contributions that may benefit facilities. However, the marketing leveraging methods used – such as branded signage and product provision – may also increase children’s exposure to food marketing. Greater monitoring and evaluation of these marketing practices are needed, especially in the context of proposed national marketing restrictions.

Sports participation is widely promoted as an important strategy to improve physical and mental health among children and youth. However, the food environments surrounding sport often contradict these health-promoting goals. Children are frequently exposed to energy-dense, nutrient-poor foods and beverages in sport-related contexts, often promoted through athlete endorsements, sports-themed packaging, video game product placements and sponsorship of professional and recreational sports^(1–6)^. These marketing practices typically involve sugary drinks, fast food and snack products that are inconsistent with dietary guidelines for children^(1,7)^. Such marketing may foster favourable perceptions of unhealthy products, particularly among young audiences, potentially creating a ‘health halo’ effect whereby products appear healthy due to their association with sport^(7,8)^. In addition, scholars have argued that the food industry often overemphasises physical activity in health messaging to ‘deflect attention from its possible role in the obesity epidemic’^(9,10)^.

Among various sport-related marketing techniques, corporate sponsorship is a common and influential strategy^(11)^. Companies provide financial or in-kind support to teams, events, athletes or venues in exchange for brand exposure^(12,13)^. Sponsorship-linked marketing can include logo placement on uniforms and equipment, signage at sporting venues, distribution of coupons or product samples to athletes and spectators and naming rights to teams or facilities^(14)^. These activities are often integrated with other marketing communications – a process referred to as ‘sponsorship leverage’ – to maximise the commercial impact of the sponsor–sponsee association^(15)^. Research shows that leveraging sponsorship increases brand recall and emotional engagement with the brand, particularly among young audiences^(15)^. For example, Cho *et al.* found that Coca-Cola’s sponsorship of the Olympic Games was associated with increased product purchases during the event, while DeGaris and West reported that soft drink sponsorship of NASCAR was linked to higher consumption among fans^(16,17)^.

In Canada, recreation and sport facilities (RSF), centres that offer spaces and programming for leisure and physical activities as well as recreational, amateur and competitive sports for children and families, represent a key setting where children encounter food marketing^(18)^. RSF often sell or promote food through food services (vending and concessions), through team fundraising or events or through corporate sponsorship, as examples. A recent study found that food marketing was significantly more frequent in RSF with any food service contracts and food sponsorship agreements, separately, than those without^(19)^. Sugar-sweetened beverage companies made up 44 % of all food service contracts; fast food and sugar-sweetened beverage brands represented more than half of all sponsors^(19)^. Little is known about the marketing rights and brand-leveraging activities embedded within these agreements, which are critical to understand how these agreements might give rise to food marketing exposures. Service contracts can shape food availability and visibility within concessions, canteens and vending operations, while sponsorship can reinforce brand exposure and normalise energy-dense, nutrient-poor foods in child-friendly environments^(20,21)^. Without evidence on the nature and extent of sponsorship- and contract-linked marketing activities in children’s sport settings, policymakers may underestimate the implications of exempting sport sponsorship from legislative restrictions.

This is of particular relevance to Canada since a private member’s bill (Bill C-252) was introduced in the Canadian Parliament in February 2022 to restrict the marketing of unhealthy food and beverages to children under the age of 13 years^(22)^. Sport sponsorship was proposed to be exempted from the legislation due to concerns that restrictions may reduce children’s access to sport^(23)^. Previous policy experience suggests that, following advertising restrictions, industries such as tobacco and alcohol increased their use of sponsorship to maintain consumer exposure^(10)^. The same may occur with food companies if sponsorship remains unregulated under new marketing policies. Bill C-252 was lost after it did not receive Royal Assent before the government session ended when a federal election was called in 2025^(24)^. Nevertheless, separate from Bill C-252, Health Canada is currently proposing changes to the Food and Drugs Regulations to prohibit the advertising of certain foods to children by food manufacturers, producers and importers, food retailers and restaurants and advertising and marketing agencies^(25)^. Ongoing research on food marketing in Canada, especially in settings that may not be within the scope of food marketing legislation and regulations, such as RSF, aims to understand the intended and unintended impacts of policy change.

This study aims to address research gaps on food marketing related to RSF by analysing the prevalence, financial value and marketing elements of food sponsorship agreements and food service contracts in Canadian RSF. Specifically, we examine whether these agreements include brand-leveraging components such as logo display, product sampling and exclusive vendor rights. By documenting the commercial integration of food companies into community sport environments, this study contributes to a better understanding of how RSF may function as a setting for children’s exposure to unhealthy food marketing and provides timely evidence to inform public health policy and nutrition education strategies.

## Method

### Participants and sampling

Using the Open Database of RSF from Statistics Canada, supplemented by recreation and sport stakeholder lists obtained from regional parks and recreation associations and internet reviews of municipality webpages and minor hockey and soccer league websites, we generated a list of 1705 potentially eligible RSF in nine provinces across Canada^(26)^. We excluded Prince Edward Island due to the lack of research staff located in the province and the small number of facilities from Prince Edward Island in Canada (<0·1 %). Eligible RSF offered organised indoor sport and recreation spaces and programming for the three most popular sports among children under 18 years in Canada (i.e. soccer, ice hockey and swimming), including ice arenas, pools and soccer fieldhouses^(27)^. We excluded seasonal recreation and sport spaces that were closed at the time of data collection (e.g. outdoor pools). We restricted our list to facilities within 150 kilometres of the most populated municipalities in each province, providing geographic spread across regions.

We randomly selected facilities by four strata according to geography (urban/rural) and sport type (ice/non-ice), until we reached our goal sample size of 120 facilities (determined *a priori* for measuring the study’s primary outcome – frequency of food marketing instances (reported elsewhere)). Ice facilities were RSF with ice surfaces used primarily for minor hockey programming; non-ice facilities had no ice surfaces. Rural RSF were defined as those located in places with fewer than 5000 people for all provinces except Ontario and Quebec, where the cut-off was fewer than 15 000 people; urban RSF were located in centres with any larger population size.

The managers/directors of 341 randomly selected RSF were contacted by phone and email up to three times to obtain consent to participate in the study. We received responses from 175 RSF, of which 137 agreed to participate (78·3 % of RSF that responded). Thirty-eight RSF declined, citing non-interest, limited resources, COVID-19 or other undisclosed reasons. Each RSF was offered a $25 prepaid e-gift card. This study was approved by institutional research ethics boards at Memorial University of Newfoundland (20220510), Public Health Ontario, Dalhousie University (2021-5850) and the Universities of Alberta (Pro00105324), Calgary (REB20-0415) and Saskatchewan (BEH 16-314).

### Data collection

Consenting RSF managers/directors were sent a link to complete an online survey via Qualtrics in July 2023 (Provo, UT) through a personalised email. Three reminders were sent to encourage the full completion of the survey. Respondents could stop and restart the survey on the same computer and browser at any time. The survey collected information on facility characteristics and the count, value and leveraging methods of food sponsorship agreements and food service contracts and was available in English and French. ‘Prevalence’ represents the proportion of RSF with current food sponsorship agreements and food service contracts, separately, and the count of sponsorship agreements and food service contracts per facility. ‘Value’ represents receiving financial donations from a sponsor or service provider for sponsorship agreements. ‘Leveraging methods’ represents active or passive marketing communications and activities such as traditional advertising, public relations activities, direct marketing, sale promotions included in sponsorship agreements and food service contracts^(15)^.

First, the survey assessed multiple facility characteristics: (i) facility size (number of sport areas; concessions; vending machines); (ii) sport type (ice/non-ice); (iii) location (urbanity; region); (iv) ownership and management models; (v) user profile (number of visitors; proportion of visitors who are children under 13 years or between 13 and 17 years; child sports teams); and (vi) facility income. A copy of the survey is available elsewhere^(19)^. With the exception of facility size, variables were measured categorically as per their response options in the survey. Respondents answered the survey in reference to their last usual fiscal year prior to the COVID-19 pandemic.

Next, we asked respondents to state the number of sponsorship agreements the RSF had with food-related and non-food-related corporations, as well as the total direct cash the RSF received from all sponsors. Sponsors were defined as outside companies or organisations that supported RSF operations through funding, materials and/or programmes and may have been advertised in and around the RSF. Respondents provided the names of the top 25 food and ‘other’ sponsors that made the greatest financial contribution in the last fiscal year. A ‘food sponsor’ was a sponsor from a food or non-alcoholic beverage company (e.g. restaurant, food or beverage brand, grocery store); an ‘other sponsor’ was a sponsor from a non-food/non-beverage company (e.g. automotive) or from an alcoholic beverage company (e.g. brewery, pub).

For the ten food sponsors that provided the greatest financial contribution in the last fiscal year, we asked RSF to report the value of any financial (direct cash) donations and the use of any financial donations from food sponsors (open-ended). Respondents were asked to select all ‘benefits’ of each food sponsorship agreement from a list previously developed by Kelly *et al.* to assess the nature and extent of sponsorship in children’s sports clubs in Australia^(4)^. These ‘benefits’ are interpreted here as leveraging methods. Participants reported the presence of the following ‘benefits’ (leveraging methods) in food sponsorship agreement: equipment for athletes; equipment for sport functions; equipment for facility vending machines; concession stands/snack bar; free or discounted products to members of programme/service/club; vouchers for products; incentive programmes (reward achievements with branded products); signage in facility areas; digital signage; logo and promotion in information pamphlet or education materials; logo or brand information on website; naming rights to facility areas (e.g. Rogers fitness centre); naming rights to facility events; sponsored events/tournaments; and fundraising. Participants could also specify any other ‘benefits’ not listed.

Finally, respondents were asked the number and names of companies with whom they had food service contracts in their RSF in the last fiscal year. Food service contracts were defined as legal agreements specifically between food and non-alcoholic beverage suppliers (e.g. Coca-Cola, PepsiCo, Tim Hortons, Aramark) and the facility to exclusively promote and/or use their products. For each contract, we asked whether the service provider included any financial support and any leveraging methods, including exclusive pouring rights; sponsored fundraising events (e.g. concerts, carnival); franchises (e.g. coffee bars, snack bars); free food or drinks, scoreboards, schedule boards and facility equipment (e.g. fridge, vending machines); decals; and labelling (e.g. stickers to highlight a product). Participants were asked to specify any other ‘benefit’ (leveraging method), if applicable.

### Statistical analysis

Analyses were run to describe the presence, value and leveraging methods of food sponsorship agreements and food service contracts. We ran descriptive analyses including calculating the median (25th percentile, 75th percentile) for continuous variables due to outliers and proportions and cross-tabulations for categorical variables. For sponsorship agreements, we calculated the proportion of RSF with sponsorship agreements, the proportion of RSF with any sponsors and the contribution of food sponsors out of all sponsors. We calculated the proportion of RSF that received financial and ‘in-kind’ benefits from any food sponsor, as well as the proportion of food sponsorship agreements that included financial and ‘in-kind’ benefits. The median financial contribution from food sponsors out of total sponsorship income per RSF was calculated, as well as the median financial contribution from food sponsors. For food service contracts, we calculated the proportion of RSF with food service contract agreements, as well as the proportion of food service contracts that included financial benefits and leveraging activities. Missing responses, as well as responses of ‘don’t know’ and ‘prefer not to say’, were excluded from the denominator in all proportion calculations. All analyses were completed using Statistical Package for the Social Sciences, Version 29 (SPSS Inc.).

## Results

Ninety-nine surveys were attempted (response rate: 73·9 %); eighty-three surveys were fully completed. A total of eighty-six surveys were included (finished at least up to the end of the food sponsorship section).

### Facility characteristics

Sixty percent of RSF were located in urban areas (*n* 52), and the same proportion were ice rinks (*n* 52). RSF were distributed across Canadian regions (British Columbia: 19·8 %, Alberta: 24·4 %, Saskatchewan: 9·3 %, Manitoba: 10·5 %, Ontario: 12·8 %, Quebec: 3·5 % and Atlantic provinces: 19·8 %). The majority of RSF were owned (82·6 %) and managed (74·4 %) by a municipality. RSF had annual incomes ranging from less than $100 000 up to $15 million; incomes between $100 000 and $500 000 were the most common (45·0 %). The median (25th percentile, 75th percentile) number of visitors was 75 000 (15 722, 255 000) per year. Children under 13 years and between 13 and 17 years made up at least one-quarter of facility visitors in 73·4 % and 50·8 % of facilities, respectively. Almost all (97·1 %) reported having sport teams, clubs or leagues for children under 18 years of age (e.g. minor ice hockey league) that used their facility in the past fiscal year. RSF had a median of 5·0 (3·0, 14·5) child sport teams, clubs or leagues that used their facility in the past fiscal year.

### Sponsorship agreements

#### Prevalence

Half of RSF (*n* 37, 50·1 %) reported having at least one sponsor of any kind (excluding twelve facilities that selected ‘don’t know’ and one that selected ‘prefer not to say’); over one-third of RSF (*n* 27, 36·5 %) had at least one food sponsor. Nine RSF had only ‘other’ sponsors, including one with a single sponsor from an alcohol company. Two RSF had food sponsors only.

RSF reported having more other sponsors (median = 5·0 (1·5, 16·5) per RSF) than food sponsors (median = 2·0 (1·0, 3·75) per RSF). Food sponsors made up 21·3 % (*n* 70) of all sponsors named by RSF. The types of food sponsors (e.g. fast food, grocery) present in RSF are reported elsewhere^(19)^.

#### Financial value

**Figure 1.**
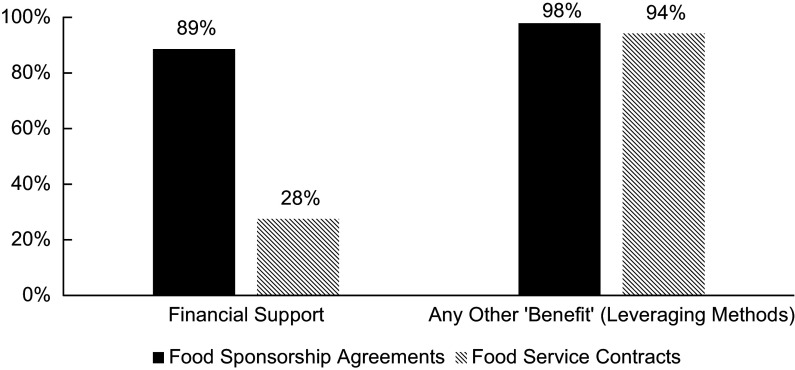
Proportion of food sponsorship agreements and food service contracts that included financial support and any other ‘benefits’ (leveraging methods).

Financial information associated with food sponsorship agreements was reported for 62·9 % (*n* 44) of food sponsors. Of these food sponsors, the majority (*n* 39, 88·6 %) provided a financial donation to RSF (Figure 1); five sponsors did not provide any funds as part of their agreement. The median annual financial donation per sponsor was $500 ($288, $1375). There were five sponsors that provided more than $10 000 per year (maximum was $60 000).

Total income from all sponsors (food and other) and income from food sponsors only were available from sixteen RSF. The median facility income from all sponsors was $9429 ($1750, $19 500). The median income from food sponsors was only $3500 ($813, $10 160). Of the RSF that received some funds from food sponsors, this income contributed a median of 25·0 % (13·9 %, 83·3 %) of their total income from sponsors.

The income from most food sponsors (65·9 %) was reportedly used to support general facility operations. For one-quarter (24·3 %) of food sponsors, the income supported the facilities’ programming. For the remaining food sponsors (10·8 %), the income was reportedly used to support the purchase of supplies or equipment.

#### ‘Benefits’ (leveraging methods)

Almost all (97·9 %) food sponsorships included at least one ‘benefit’ or leveraging method (Figure 1). The majority received physical and/or digital signage (*n* 42, 64·6 %) (Table 1). Logos and branding for websites or paper materials, food services through vending or concession, equipment and food provision were the next most common (Table 1).

**Table 1.** ‘Benefits’ (leveraging methods) included in food sponsorship agreements (*n* 65 sponsors)

‘Benefit’ (leveraging method)	Food sponsors^*^	
	*n*	%
Branded signage	42	64·6 %
Signs in facility areas	40	
Digital signage	4	
Logos, promotions and/or brand information	17	26·2 %
Logo or brand information on website	12	
Logo and promotion in information pamphlets or education materials	9	
Food services	16	24·6 %
Vending machines	10	
Concession stands/snack bar	8	
Equipment	12	18·5 %
Equipment for facility	12	
Equipment for sport functions	1	
Food provision	11	15·4 %
Free or discounted products to members of programme/service/club	5	
Vouchers for products	4	
Other food provision for facility	2	
Sponsored events/tournaments	9	13·8 %
Naming rights	6	9·2 %
Naming rights to facility areas	5	
Naming rights to facility events	2	
Incentive programmes (reward achievements with branded products)	2	2·9 %
Other	1	1·5 %
None	2	2·9 %

* Excluding *n* 2 ‘don’t know’, *n* 1 ‘prefer not to say’.

**Table 2.** Leveraging methods included in food service contracts (*n* 69 contracts)

Leveraging methods	Food service contracts	
	*n*	%
Facility equipment*	36	52·2 %
Exclusive food or beverage rights	26	37·7 %
Decals	8	11·6 %
Sponsored fundraising events^†^	6	8·7 %
Labelling^‡^	6	8·7 %
Sport equipment^§^	5	6·2 %
Free food or drinks	4	5·8 %
Revenue from % of sales	4	5·8 %
Franchises^||^	3	4·3 %

*For example, vending machines, fridges, ^†^For example, concerts, carnivals. ^‡^For example, stickers to highlight a product. ^§^For example, scoreboard, schedule board. ^||^For example, coffee bars, snack bars.

### Food service contracts

#### Prevalence

Food service contracts were common across RSF (*n* 42, 65·6 %) (excluding fifteen facilities that select ‘don’t know’ and eight that selected ‘prefer not to say’). A total of seventy food service contracts were listed. RSF had a median of 2·0 (1·0, 2·0) food service contracts. The types of food service providers (e.g. sugar-sweetened beverage manufacturers) present in RSF are reported elsewhere^(19)^.

#### Financial value

Financial donations were provided in one-quarter of food service contracts (*n* 19, 27·5 %) (Figure 1). Commission from sales was reported to be in four contracts (Table 2).

#### ‘Benefits’ (leveraging methods)

Non-financial ‘benefits’ or leveraging methods were identified in almost all food service contracts (*n* 65, 94·2 %) (Figure 1). The most common leveraging method was the provision of facility equipment (e.g. vending machines, fridges) (*n* 36, 52·2 % of contracts). Approximately one-third of contracts were reported to have exclusivity over food or beverage provision (*n* 26), despite this being part of the given definition of food service contracts in the survey (Table 2).

## Discussion

This study aimed to investigate the prevalence, value and leveraging methods of food sponsorship agreements and food service contracts in RSF in Canada. One-third of RSF sampled had at least one food sponsorship agreement; approximately two-thirds of facilities had at least one food service contract. Almost all food sponsorship agreements and food service contracts included product or brand-leveraging methods in the form of ‘in-kind’ donations that may have variable benefits for the sponsor *versus* the RSF. For sponsorship agreements, the most common leveraging method appeared to favour the sponsor (e.g. signage, logos/brands); a smaller proportion of leveraging methods from sponsors may benefit the RSF (e.g. food service provision, equipment, food provision). The most common leveraging method in food service contracts was equipment donation (which may benefit the RSF), followed by food or beverage provision exclusivity (which may provide a greater benefit to the service provider). While financial donations can help support children’s sport activities, the impact of so-called ‘benefits’ of sponsorship agreements via in-kind sponsorship donations, such as branded items, naming rights, etc., is likely to be of limited benefit to children. In contrast, such marketing communications that support brand leveraging have clearer benefits for corporations in building brand awareness early with young consumers. Previous research found that the majority of food sponsors and food service providers in RSF were linked to at least one food marketing instance and contributed approximately one-fifth of food marketing instances within RSF^(19)^, which may be explained by the leveraging methods identified here.

Most food sponsorship agreements had a financial component. Only about one-quarter of food service contracts included financial donations to the facility. Food sponsorship agreements made up approximately 21 % of all sponsorship agreements and provided 25 % of the total amount provided from all sponsors. Said another way, food sponsorship agreements made up one in five of all sponsorship agreements and provided $1 out of every $4 of total sponsorship dollars. In previous research, we found a considerably higher median number of marketing instances associated with sugar-sweetened beverage sponsors (12 (9·0, 27·0)), compared with all other food and beverage sponsors (1·0 (1·0, 3·0)). The financial donations from sponsors should be weighed against their potential contribution to unhealthy food and beverage marketing exposures. More research should evaluate the financial contribution by type of food company.

Debates about the appropriateness of sport sponsorship are complex, in part because of a fear of financial strain on centres and sports organisations resulting from the rejection of sponsorships, as well as a perceived notion by sports administrators that there is a broad public acceptance of this type of sponsorship in sport. Barriers to improving food environments and sponsorship in RSF are largely centred on a fear of revenue loss, perceived consumer discontent at the loss of preferred unhealthy food options and a lack of organisational capabilities and resources to generate change^(21)^. On the other hand, health-promoting policies, managerial support for change, community engagement and education and capacity building can facilitate successful improvements to RSF food environments^(21)^. More research is needed to understand how these facilitators and barriers relate to food sponsorship agreements, food service contracts and food marketing outcomes.

It is well recognised that sponsorship has evolved from a philanthropic activity to a brand engagement, consumer-oriented marketing communications platform; however, the effectiveness and value of sponsorship are poorly understood^(12,28)^. The impacts of sponsorship appear to be generated from leveraging methods where the sponsorship is integrated with other marketing activities^(12)^. It is crucial to scrutinise the whole mix of marketing activities used in recreation and sport, including but not limited to sponsorship, when examining food marketing in children’s settings. More research is needed to understand the decision-making and management of food-related domains in recreation and sport settings, including sponsorship, food provision and marketing^(29)^.

The financial value of sponsors for RSF cannot be ignored; however, the contribution of cash from food sponsors may not be significant^(4)^. Kelly *et al.* found that the majority of Australian sports clubs received less than one-quarter of their overall income from sponsorship^(4)^. Our study found that food sponsorship agreements contributed to one-quarter of all sponsorship income, which is only one source of revenue for the RSF. It may be possible for RSF to replace their existing food sponsors (and their associated financial donations) with other sponsors. However, since RSF had numerous other sponsors, it is unclear how close the current number of other sponsors is to its ceiling. Research on food sponsorship in recreation and sport in Canada is only just emerging. Pauze *et al.* published a pilot study assessing sponsorship of sport leagues (regional and national sporting organisations, not physical facilities) in Ottawa, Canada, for the five most popular sports (soccer, swimming, hockey, basketball and baseball) for Canadian children^(30)^. Pauze *et al.* reported that approximately 40 % of sport leagues had some type of food sponsor^(30)^. Food sponsors represented a comparable proportion of total sponsors (16 %) in the study by Pauze *et al.* ^(30)^. Similarly, a study of sports clubs conducted in Australia also reported that food sponsors represented 17 % of total sponsors^(4)^.

Food service contracts have not been well studied in RSF, despite potentially acting as a barrier or facilitator to healthy food environments^(31)^. Exclusive pouring rights contracts with beverage companies are alarming in children’s settings as they promote their products through availability and branding and contribute to brand loyalty in young consumers^(32)^. Nestle warned that exclusive pouring rights in schools have clear benefits to the beverage companies with questionable impacts on schools and that some contracts may result in schools ‘pushing’ soft drinks to their staff and students, so the school receives additional financial donations for greater purchasing from vending machines^(32)^. Similar effects may be expected within RSF. Research in Canada has shown that the foods and beverages available in vending machines are healthier when the vending provider has a contract with the facility that includes a minimum nutrition criterion, compared with a contract without nutrition criteria^(20)^. The criteria included in food service contracts could expand to not only stipulate the nutritional quality of products but also restrict marketing activities such as exclusivity, branding on vending machine facades or equipment, fundraising and giveaways. Comprehensive nutrition policies in RSF that address both availability and marketing can positively impact the healthfulness of foods and beverages purchased at concessions in these facilities^(33)^.

### Strengths and limitations

This study was conducted during the COVID-19 pandemic, which affected the capacity of RSF to participate due to facility closures, operational changes and staff lay-offs. Although we conducted random sampling within our sampling frame, self-selection bias may have impacted the participation of RSF due to the opt-in nature of the study. Nonetheless, our sample provided diverse representation across Canada and sport types. We had high participation rates among facilities that responded to our invitation and good survey completion from all facilities in most regions.

The number of respondents who reported they ‘don’t know’ or ‘prefer not to say’ the number of food sponsorship agreements or food service contracts made up 15–23 % of respondents, respectively. This uncertainty may exist because of the multiple operational areas in RSF related to food; depending on how the RSF operates, the respondent may not have been informed about all aspects of food, including the contracts or agreements asked about in the survey. We encouraged respondents to seek out answers to questions they were unsure about, but it may have been too burdensome for them to do so. Future work should attempt to more comprehensively collect data on legal agreements, such as through phone interviews or document reviews, to avoid missing data due to a lack of individual knowledge.

Further, future efforts should attempt to examine other sponsorship agreements in the recreation and sport setting, including those with sports teams/clubs/leagues. We were unable to include data on sponsorship from sport teams/clubs/leagues as we had 45 % of data missing or from respondents who were unaware of the sponsors of sport teams/clubs/leagues that used their facilities. The current project underestimates the breadth of leveraging methods used by sport sponsors. Sports clubs/leagues may have their own sponsorship agreements^(30)^, with different marketing leveraging methods, including ones that may more actively engage children and youth (e.g. jerseys, vouchers, rewards). Marketing from sports clubs/leagues may reinforce the sponsorship-linked (contract-linked) marketing present in RSF.

### Conclusion

This study explored food sponsorship agreements and food service contracts in a diverse sample of RSF in Canada. Both sponsorship agreements and food service contracts were reported to include leveraging methods that could be considered sponsorship-linked (contract-linked) marketing. Such marketing leveraging methods may provide various benefits not only to the facility through operations and programming but also to the sponsor or contract provider through increased product and brand visibility. Financial donations were more common through sponsorship agreements than through food service contracts, but their contribution to facilities’ overall operations may be overvalued.

There are many commercial marketing activities included in these sponsorship agreements and food service contracts that may be disguised as ‘in-kind’ donations, including free branded equipment or coupons and vouchers for food. The contribution of these leveraging methods is a potential source of unhealthy food and beverage marketing to children in RSF. With the potential for forthcoming restrictions on food marketing to children in Canada that may allow continued sports sponsorship in children’s settings, ongoing monitoring of food marketing environments in recreation and sport in Canada will be critical to evaluating the effectiveness of a national policy to protect children from unhealthy food marketing and identify any unintended impacts on the recreation and sport sector.
